# Perceived Stress, Resilience, and Wellbeing in Seasoned Isha Yoga Practitioners Compared to Matched Controls During the COVID-19 Pandemic

**DOI:** 10.3389/fpubh.2022.813664

**Published:** 2022-07-29

**Authors:** Preeti Upadhyay, Shilpa Narayanan, Tanvi Khera, Lauren Kelly, Pooja A. Mathur, Akshay Shanker, Lena Novack, Ruth Pérez-Robles, Kim A. Hoffman, Senthil Kumar Sadhasivam, Balachundhar Subramaniam

**Affiliations:** ^1^Beth Israel Deaconess Medical Center, Boston, MA, United States; ^2^Oregon Health and Science University-Portland State University School of Public Health, Portland, OR, United States; ^3^Riley's Children's Hospital, Indiana University, Bloomington, IN, United States

**Keywords:** yoga, meditation, perceived stress, Isha Foundation, wellbeing

## Abstract

**Background:**

Yoga practices, including breathing, meditation, and posture protocols (asanas), have been shown to facilitate physical and mental wellbeing.

**Methods:**

Seasoned yoga practitioners were recruited from the Isha Foundation. Recruitment of the comparison group was achieved using snowball sampling and were not yoga practitioners. Participants in the non-yoga group were randomized to a 3-min Isha practice or a comparator group asked to perform 15-min of daily reading. Participants completed a series of web-based surveys (REDCap) at baseline, 6, and 12 weeks. These surveys include validated scales and objective questions on COVID-19 infection and medical history. The validated questionnaires assess for: perceived stress (PSS), mood states [anxiety and depression (PHQ-4), joy (DPES-Joy subscale)], mindfulness attention and awareness (MAAS), resilience (BRS), mental wellbeing (WEMWBS) and recovery from traumatic event (PTGI). Weekly activity diaries were employed as a tool for collecting compliance information from study participants. Perceived stress scale scores were identified as primary outcome for this study.

**Findings:**

The median Perceived Stress Scale (PSS) score for the yoga practitioners compared to the active and placebo comparators was significantly lower at all time-points: baseline: 11 [IQR 7–15] vs. 16 [IQR 12–21] in both the active and placebo comparators (*p* < 0.0001); 6 weeks: 9 [IQR 6–13] vs. 12 [IQR 8–17] in the active comparator and 14 [IQR 9–18] in the placebo comparator (*p* < 0.0001); and 12 weeks: 9 [IQR 5–13] vs. 11.5 [IQR 8–16] in the active comparators and 13 [IQR 8–17] in the placebo comparator (*p* < 0.0001). Among the randomized participants that were compliant for the full 12 weeks, the active comparators had significantly lower median PSS scores than the placebo comparators 12 weeks [10 (IQR 5–14) vs. 13 (IQR 8–17), *p* = 0.017]. Further, yoga practitioners had significantly lower anxiety at all three-time points (*p* < 0.0001), lower depression at baseline and 6 weeks (*p* < 0.0003), and significantly higher wellbeing (*p* < 0.0001) and joy (*p* < 0.0001) at all three-time points, compared to the active and placebo comparator groups.

**Interpretation:**

The lower levels of stress, anxiety, depression, and higher level of wellbeing and joy seen in the yoga practitioners compared to the active and placebo comparators illustrate the impact of regular yoga practices on mental health even during the pandemic.

**Trial Registration:**

ClinicalTrials.gov, identifier: NCT 04498442.

## Introduction

The global toll of COVID-19 on physical and mental health has been severe, as the disease continues to disrupt lives and impact wellbeing (1, 2). Research has already documented that psychological distress (3, 4), anxiety (5), sleep disturbances (6), greater feelings of isolation (7), and problematic substance use have increased as a result of the pandemic (8). In addition, perceived stress has been shown to accompany COVID-19 infection and treatment (9). Adopting strategies to maintain or increase mental, emotional, and physical health during these difficult times will enable greater resilience in individuals as we begin to emerge from the pandemic (10, 11). In fact, the US Centers for Disease Control has emphasized the importance of managing stress during the pandemic time and avoiding maladaptive behaviors to cope with stress and anxiety (9).

Yoga practices, including breathing, meditation, and posture protocols (asanas), have been shown to facilitate enhanced physical and mental wellbeing (12, 13). Enhanced wellbeing is achieved through improvements in the modulation of the autonomic nervous system (14), improved sleep quality (15), and immunity (16), and reductions in stress (17, 18), anxiety (19), and depression (20, 21) in regular yoga practitioners. In a meta-analysis of 47 trials, researchers found evidence that meditation reduced multiple negative dimensions of psychological stress such as anxiety and depression (22). Similarly, in a study of yogic breathing practices, functional magnetic resonance imaging (fMRI) showed significantly decreased states of anxiety and negative affect and modulation of activity in brain regions involved in emotional processing, attention, and awareness (23). A significant decrease in perceived stress after a single yoga class (17) and after an 8-week course (18) suggests that yoga has both immediate and longer-term impacts on perceived stress during continued yoga practice. The Centers for Disease Control and Prevention reports that an increasing number of adults are practicing yoga and meditation to enhance wellbeing (24).

The Isha Foundation (25), an international school of yoga, teaches yogic practices designed to meet individual needs and improve wellbeing. The Isha Foundation's Inner Engineering course including seven online modules, and a 1-day in-person program were offered in the traditional modality before the pandemic and online-only during the pandemic. We enrolled 8,519 participants (6,892 regular Inner Engineering practitioners vs. 2,344 age, gender, and zip code matched controls) to test the program's effectiveness. We evaluated the stress and wellbeing of participants at three different times during this pandemic. Participants in the non-yoga group were not yoga practitioners and were randomized into either the *active comparator arm* or the *placebo comparator arm*. We randomized the non-yoga group to a simple 3-min breathing practice group and an active reading control group to assess the effect on perceived stress. We hypothesized that those undertaking the yogic practices would (a) have the least amount of perceived stress and (b) report higher levels of wellbeing than the control [specifically, the placebo comparator group (i.e., reading group)] over the study period of May 2020 to September 2020.

## Methods

We designed the study to comply with the then COVID-19 precautions imposed by federal and state governments in the U.S. Beth Israel Deaconess Medical Center Institutional Review Board approved this study.

### Recruitment and Intervention

The methodology paper detailing the study has been published elsewhere (26). Briefly, seasoned Isha yoga practitioners were recruited by social media, websites, flyers, word of mouth, and email announcements from the Isha Foundation. Participants consented to study participation *via* REDCap. The second stage of recruitment used snowball sampling (27). Yoga practitioners were requested to nominate two friends or colleagues who did not practice yoga within the last month as age, gender, and zip-code matched controls (preferably from the same neighborhood). The study team reached out to those nominated and obtained REDCap consent from those who chose to enroll.

Non-meditator controls were randomized to an *active comparator arm* or the *placebo comparator arm*. Those in the *active comparator arm* were taught a 3-min yoga practice, Simha Kriya, which involved a specific breathing practice with rapid, deep breathing and breath retention designed during the pandemic to improve the pulmonary function. It was perceived as useful by 77% of healthcare workers participating in a study conducted at MD Anderson (Houston, TX) during the pandemic's peak (28). These participants were asked to perform Simha Kriya using a web-based application twice per day. Those randomized to the *placebo comparator arm* performed either reading activities or remained idle for 15-min a day throughout the study period.

Respondents in the observational arm (seasoned yoga practitioners) continued their usual yoga practices. On an average, seasoned yoga practitioners reported to have ~5.6 years (SD: ± 7.2) of practice experience. Their average session duration was reported to be 6.6 h (SD: ± 5.7) each day. The expertise of yoga in seasoned practitioners varied greatly. Their expertise ranged from practitioners who recently completed inner engineering online course and performed simple yoga practices for 30-min each day to highly motivated practitioners with 6 h or more of dedicated meditation and yoga practices.

### Data Collection and Measures

Participants completed a series of web-based surveys (REDCap) at baseline, 6, and 12 weeks. Data collection included weekly activity diaries, medical history, and eight validated neuropsychological scales assessing stress (Perceived Stress Scale, PSS), anxiety and depression (PHQ-4), joy predisposition (DPES-Joy Subscale), mindfulness awareness (MAAS), resilience (BRS), mental wellbeing (WEMWBS), and post-traumatic growth (PTGI). PSS is defined as the primary outcome, while BRS was the key secondary outcome.

### Statistical Analysis

Descriptive statistics are presented to summarize the data. Continuous data were presented as median [interquartile range] after confirming with the Shapiro-Wilk test that data did not follow a normal distribution. Differences within groups between baseline and 6 weeks and 6 and 12 weeks were assessed with a Wilcoxon Signed-Rank test for paired data. Differences between two groups were assessed with a Wilcoxon Rank-Sum test and between three groups with a Kruskal–Wallis test. Categorical data were presented as frequencies and percentages and assessed with chi-square or Fisher's exact test, as appropriate.

All primary analyses were assessed using intention-to-treat principles. Further, differences between groups for the primary and key secondary outcomes (PSS and BRS) were also assessed using a Poisson Regression model with scaled deviance to adjust for potential confounding by region, employment status, and age. SAS 9.4 (SAS Institute Inc., Cary, NC) was used for all analyses with two-sided *p* < 0.05 considered statistically significant.

## Results

**Figure 1:**
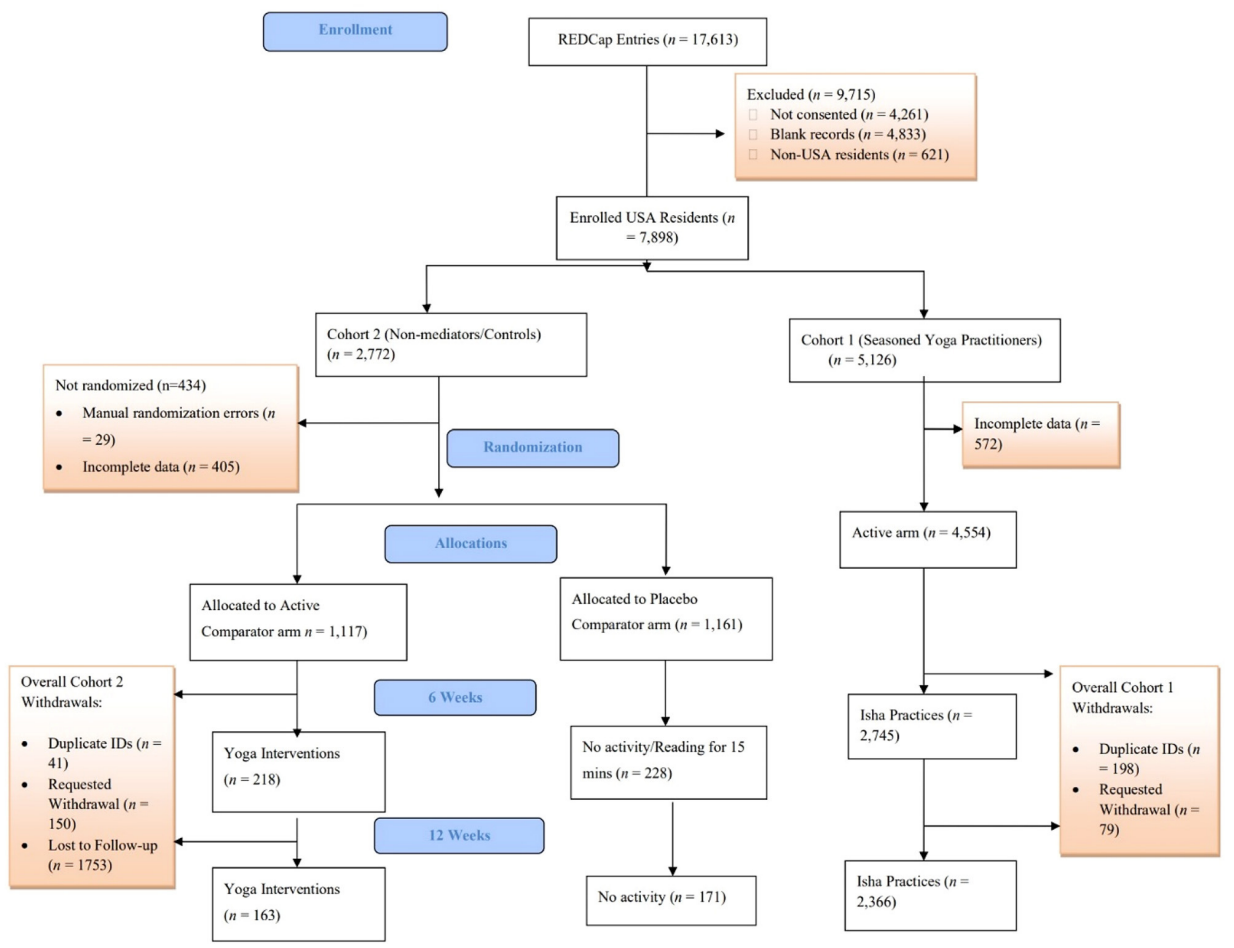
CONSORT flow diagram.

Of the 8,519 participants that agreed to participate, 6,892 participants were included in the baseline analysis because they were from the United States and had at least partially complete data. Of these, there were 1,177 active comparators, 1,161 placebo comparators, and 4,554 yoga practitioners. At 6 weeks, 218 (18.5%) of the active comparators, 228 (19.6%) of the placebo comparators, and 2,745 (60.3%) of the yoga practitioners remained in the study. At 12 weeks, these numbers reduced to 163 (13.8%), 171 (14.7%), and 2,366 (52%), respectively (Figure 1).

### Baseline Characteristics

**Figure 2:**
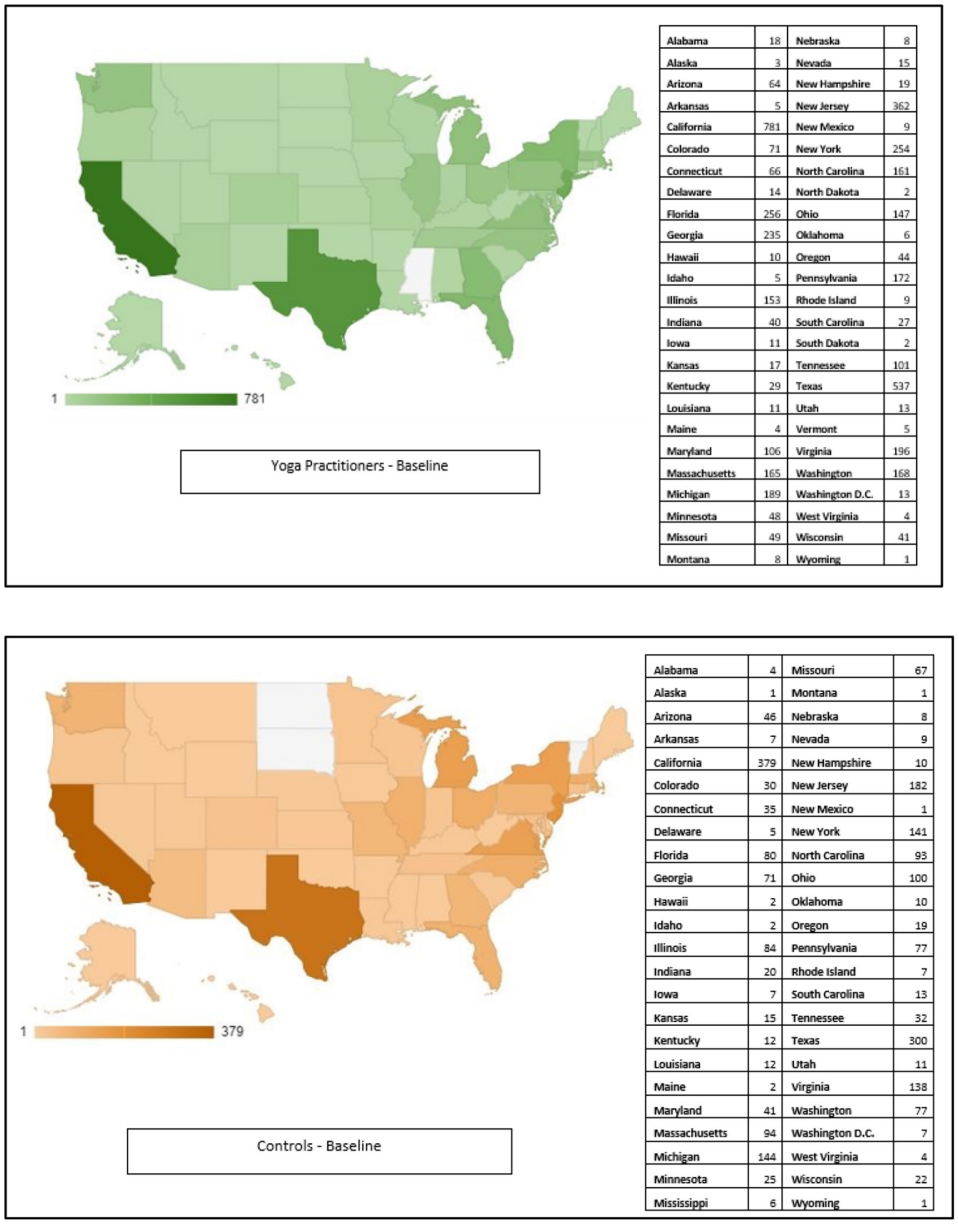
Geo-charts (baseline only).

Around 57% of participants in each group were females with mean ages ranging from 42 to 45, and more than half of each group having a higher education than a Bachelor's Degree. Sixty-four percentage of active and placebo comparators were employed full-time and 14% part-time compared to 57 and 20%, respectively, among the yoga practitioners (Table 1). There were also significant differences in the regions that the groups reside in. Around 20% of the comparator groups were from the Midwest, but only 15% among yoga practitioners. These characteristics that were found to be significantly different were adjusted for in the primary analyses (Refer to Figure 2 for geo-distribution at baseline, remaining charts available in Supplementary Materials).

**Table 1:** Demographic characteristics.

	**Active** **comparators** **(*n* = 1,177)**	**Placebo** **comparators** **(*n* = 1,161)**	**Yoga** **practitioners** **(*n* = 4,554)**	***P*** **-Value**
**Gender, No. (%)**				
Female	679 (57.7)	666 (57.4)	2,631 (57.8)	1.0
Male	497 (42.2)	494 (42.6)	1917 (42.1)	
Other	1 (0.1)	1 (0.1)	6 (0.1)	
**Age**				<0.0001
Median (IQR)	42 (33, 50)	41 (33, 50)	43 (36, 52)	
Mean (SD)	42.5 (13.0)	42.3 (12.8)	44.6 (11.7)	
**Educational qualifications, No. (%)**				0.20
Less than bachelor's degree	141 (12.0)	123 (10.6)	491 (10.8)	
Bachelor's degree	390 (33.1)	370 (31.9)	1,391 (30.5)	
Higher than bachelor's degree (Master's, Professional, Ph.D)	646 (54.9)	668 (57.5)	2,672 (58.7)	
**Employment status, No. (%)**				<0.0001
Employed full time	754 (64.0)	743 (64.0)	2,603 (57.2)	
Employed part time (self-employed, contingent worker)	163 (13.9)	160 (13.8)	928 (20.4)	
Not employed/Laid off	113 (9.6)	122 (10.5)	493 (10.8)	
Retired	53 (4.5)	64 (5.5)	252 (5.5)	
Other (disabled, student, military service)	94 (8.0)	72 (6.2)	278 (6.1)	
**Region, No. (%)**				<0.0001
Midwest	242 (20.6)	232 (20.0)	692 (15.2)	
North East	266 (22.6)	300 (25.9)	1,152 (25.3)	
South East	218 (18.5)	234 (20.2)	1,010 (22.2)	
South West	193 (16.4)	152 (13.1)	605 (13.3)	
West	257 (21.9)	240 (20.7)	1,092 (24.0)	
Unknown	1 (0.1)	3 (0.3)	3 (0.1)	

### Primary Outcome—Perceived Stress During the Pandemic (PSS Scores)

**Figure 3:**
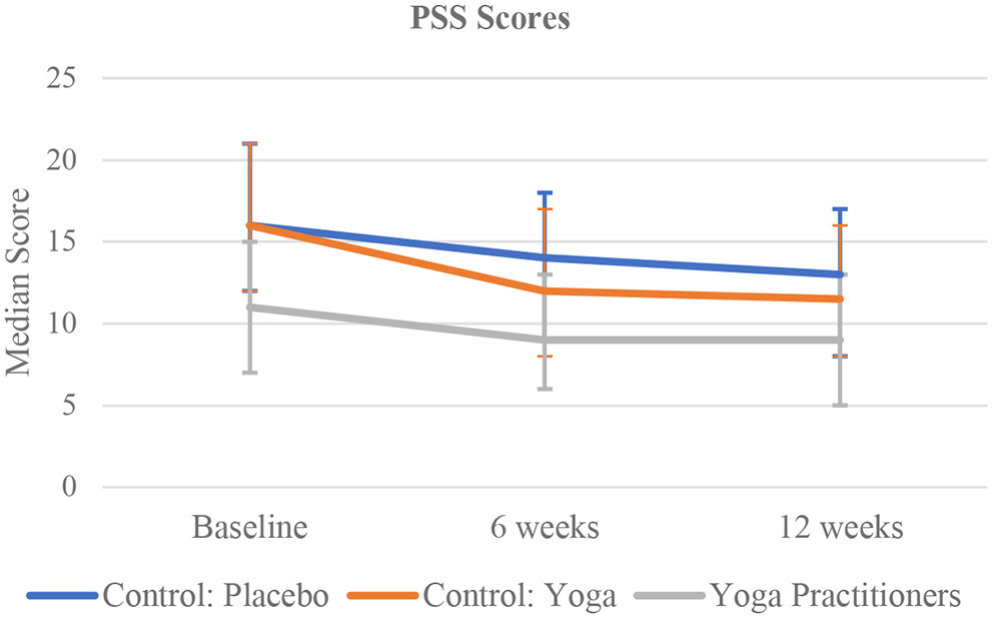
Time trend comparison of PSS Scores in the three study group at all three time points.

At baseline, 6, and 12 weeks, yoga practitioners had significantly lower PSS scores than active and placebo comparator groups (Figure 3).

The median PSS score for the yoga practitioners compared to the active and placebo comparators was significantly lower at all time-points: baseline: 11 [IQR 7–15] vs. 16 [IQR 12–21] in both the active and placebo comparators (*p* < 0.0001); 6 weeks: 9 [IQR 6–13] vs. 12 [IQR 8–17] in the active comparator and 14 [IQR 9–18] in the placebo comparator (*p* < 0·0001); and 12 weeks: 9 [IQR 5–13] vs. 11.5 [IQR 8–16] in the active comparators and 13 [IQR 8–17] in the placebo comparator (*p* < 0.0001) (Table 2). Adjusting for age, region, and employment status, the yoga practitioners, had a significantly lower PSS scores compared to the placebo comparators [31% reduction (R.R. 0.69 (95% CI 0.67–0·71)) at baseline, 30% reduction (R.R. 0.70 (95% CI 0.65–0.75)) at 6 weeks, and 29% reduction (R.R. 0.71 (95% CI 0.65–0·77)) at 12 weeks]. In the adjusted analysis, the active comparators did not have significantly different PSS scores compared to the placebo comparators at any time point (Refer to Supplementary Table A for further details).

**Table 2:** PSS scores—primary outcome (primary outcome for the 3 groups at all 3 time points).

	**Baseline**				**Week 6**				**Week 12**			
	**Active** **comparators** **(*n* = 1,177)**	**Placebo** **comparators** **(*n* = 1,161)**	**Yoga** **practitioners** **(*n* = 4,554)**	***P*** **-Value**	**Active** **comparators** **(*n* = 218)**	**Placebo** **comparators** **(*n* = 228)**	**Yoga** **practitioners** **(*n* = 2,745)**	***P*** **-Value**	**Active** **comparators** **(*n* = 163)**	**Placebo** **comparators** **(*n* = 171)**	**Yoga** **practitioners** **(*n* = 2,366)**	***P*** **-Value**
PSS score, median (IQR)	(*n* = 1,176)^a^	(*n* = 1,160)	(*n* = 4,552)	<0.0001	(*n* = 207)	(*n* = 222)	(*n* = 2,658)	<0.0001	(*n* = 146)	(*n* = 157)	(*n* = 2,108)	<0.0001
	16 (12, 21)	16 (12, 21)	11 (7, 15)		12 (8, 17)	14 (9, 18)	9 (6, 13)		11.5 (8, 16)	13 (8, 17)	9 (5, 13)	

a *(n = X) represents the number of patients with PSS scores in the specific group at the specific time point*.

### Within the Group Change in PSS Score

There was a statistically significant difference in median PSS scores between baseline and 6 weeks among all groups. The active comparators had a two-unit [IQR −5–1] decrease (*p* < 0.0001), the placebo comparators had a two-unit [IQR −5–1] decrease (*p* < 0.0001), and the yoga practitioners had a zero-unit [IQR −3–2] change (*p* < 0.0001). We also found a significant difference when comparing these median changes between the three groups (*p* < 0.0001). There were no significant differences in PSS scores between 6 and 12 weeks for any of the groups.

### Key Secondary Outcome—Brief Resilience Scores

The median BRS score at baseline for both the active comparators and placebo comparators was 2 [IQR 1.8–2.3] compared to 2 [IQR 1.8–2.2] for the yoga practitioners (*p* < 0.0001). There were no significant differences between median BRS scores at 6 or 12 weeks. There were also no significant differences between the BRS scores of the groups when adjusted for age, region, and employment status.

### Other Secondary Outcomes

**Figure 4:**
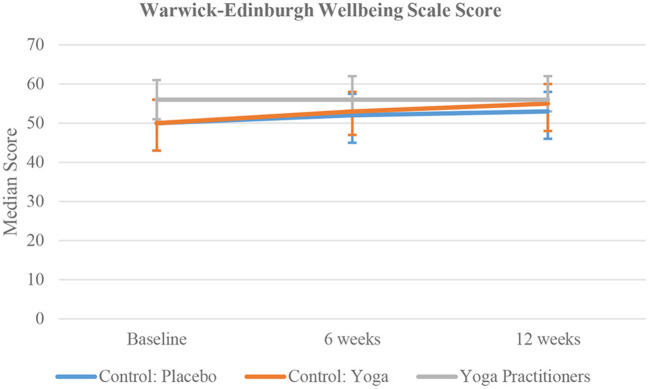
Time trend comparison of WEMWBS Score in the three study group at all three time points.

Based on the PHQ-4 scores, yoga practitioners had significantly lower anxiety at all three-time points (*p* < 0.0001), lower depression at baseline and 6 weeks (*p* < 0.0003), and significantly higher wellbeing (*p* < 0.0001) (Figure 4) and joy (*p* < 0.0001) at all three-time points, compared to the active and placebo comparator groups.

The results were analyzed for compliant participants only. At baseline, measures of negative affect (stress, anxiety, depression) and positive affect (wellbeing, joy, resilience) and mindfulness were comparable between these two groups (Refer to Table 3 for further details).

**Table 3:** Secondary outcomes (secondary outcomes for the 3 group at all 3 time points).

	**Baseline**				**Week 6**				**Week 12**			
	**Active** **comparators** **(*n* = 1,177)**	**Placebo** **comparators** **(*n* = 1,161)**	**Yoga** **practitioners** **(*n* = 4,554)**	***P*** **-Value**	**Active** **comparators** **(*n* = 218)**	**Placebo** **comparators** **(*n* = 228)**	**Yoga** **practitioners** **(*n* = 2,745)**	***P*** **-Value**	**Active** **comparators** **(*n* = 163)**	**Placebo** **comparators** **(*n* = 171)**	**Yoga** **practitioners** **(*n* = 2,366)**	***P*** **-Value**
**Measures of negative affect** ^a^												
PHQ anxiety score, No. (%)	(*n* = 1,173)^d^	(*n* = 1,156)	(*n* = 4,547)	<0.0001	(*n* = 204)	(*n* = 218)	(*n* = 2,654)	<0.0001	(*n* = 144)	(*n* = 154)	(*n* = 2,088)	<0.0001
None	982 (83.7)	958 (82.9)	4,314 (94.9)		182 (89.2)	198 (90.8)	2,572 (96.9)		133 (92.4)	140 (90.9)	2,039 (97.7)	
Mild	154 (13.1)	162 (14.0)	192 (4.2)		17 (8.3)	16 (7.3)	76 (2.9)		10 (6.9)	13 (8.4)	44 (2.1)	
Moderate	37 (3.2)	36 (3.1)	41 (0.9)		5 (2.5)	4 (1.8)	6 (0.2)		1 (0.7)	1 (0.7)	5 (0.2)	
PHQ depression score, No. (%)	(*n* = 1,173)	(*n* = 1,156)	(*n* = 4,547)	<0.0001	(*n* = 204)	(*n* = 218)	(*n* = 2,654)	0.0003	(*n* = 144)	(*n* = 154)	(*n* = 2,088)	0.051
None	999 (85.2)	993 (85.9)	4,224 (92.9)		185 (90.7)	201 (92.2)	2,541 (95.7)		135 (93.8)	144 (93.5)	2,012 (96.4)	
Mild	159 (13.6)	145 (12.5)	294 (6.5)		17 (8.3)	16 (7.3)	106 (4.0)		9 (6.3)	10 (6.5)	74 (3.5)	
Moderate	15 (1.3)	18 (1.6)	29 (0.6)		2 (1.0)	1 (0.5)	7 (0.3)		0 (0.0)	0 (0.0)	2 (0.1)	
**Measures of positive affect** ^b^												
Warwick-Edinburgh wellbeing	(*n* = 1,169)	(*n* = 1,146)	(*n* = 4,539)	<0.0001	(*n* = 195)	(*n* = 216)	(*n* = 2,623)	<0.0001	(*n* = 139)	(*n* = 152)	(*n* = 2,053)	<0.0001
scale score, median (IQR)	50 (43, 56)	50 (43, 56)	56 (51, 61)		53 (47, 58)	52 (45, 57.5)	56 (52, 62)		55 (48, 60)	53 (46, 58)	56 (53, 62)	
DPES score, median (IQR)	(*n* = 1,175)	(*n* = 1,154)	(*n* = 4,548)	<0.0001	(*n* = 200)	(*n* = 217)	(*n* = 2,650)	<0.0001	(*n* = 144)	(*n* = 153)	(*n* = 2,079)	<0.0001
	5 (4.2, 5.7)	5 (4.3, 5.7)	5.5 (4.8, 6.2)		5 (4.5, 5.8)	5 (4.2, 5.8)	5.7 (5, 6.2)		5.2 (4.4, 5.9)	5 (4.3, 5.7)	5.7 (5, 6.2)	
BRS score, median (IQR)	(*n* = 1,169)	(*n* = 1,151)	(*n* = 4,542)	<0.0001	(*n* = 198)	(*n* = 217)	(*n* = 2,639)	0.21	(*n* = 140)	(*n* = 153)	(*n* = 2,064)	0.20
	2 (1.8, 2.3)	2 (1.8, 2.3)	2 (1.8, 2.2)		2 (1.8, 2.2)	2 (1.8, 2.2)	2 (1.8, 2.2)		2 (1.8, 2.2)	2 (2, 2.2)	2 (1.8, 2.2)	
**Mindfulness scores** ^c^												
MAAS score, median (IQR)	(*n* = 1,170)	(*n* = 1,153)	(*n* = 4,545)	<0.0001	(*n* = 200)	(*n* = 217)	(*n* = 2,642)	<0.0001	(*n* = 143)	(*n* = 153)	(*n* = 2,067)	<0.0001
	4.2 (3.4, 5)	4.2 (3.2, 5)	4.8 (4, 5.2)		4.6 (3.8, 5.2)	4.4 (3.6, 5.2)	4.8 (4.2, 5.4)		4.8 (4, 5.4)	4.6 (3.6, 5.2)	5 (4.2, 5.4)	
**COVID positive participants only**												
Post-Traumatic growth	(*n* = 5)	(*n* = 7)	(n=20)	0.28	(*n* = 1)	(*n* = 1)	(*n* = 12)	0.62	(*n* = 1)	(*n* = 3)	(*n* = 26)	0.68
inventory score, median (IQR)	65 (11, 68)	63 (45, 84)	72.5 (53.5, 86.5)		56 (56, 56)	54 (54, 54)	78.5 (52, 83.5)		48 (48, 48)	73 (28, 76)	72.5 (48, 81)	

a *Decline in scores suggests successful impact of meditation practices,*

b *Increase in scores suggests successful impact of meditation practices,*

c *increase in scores suggests successful impact of meditation practices,*

d *(n = X) represents the number of patients with this score in the specific group at the specific time point*.

**Figure 5:**
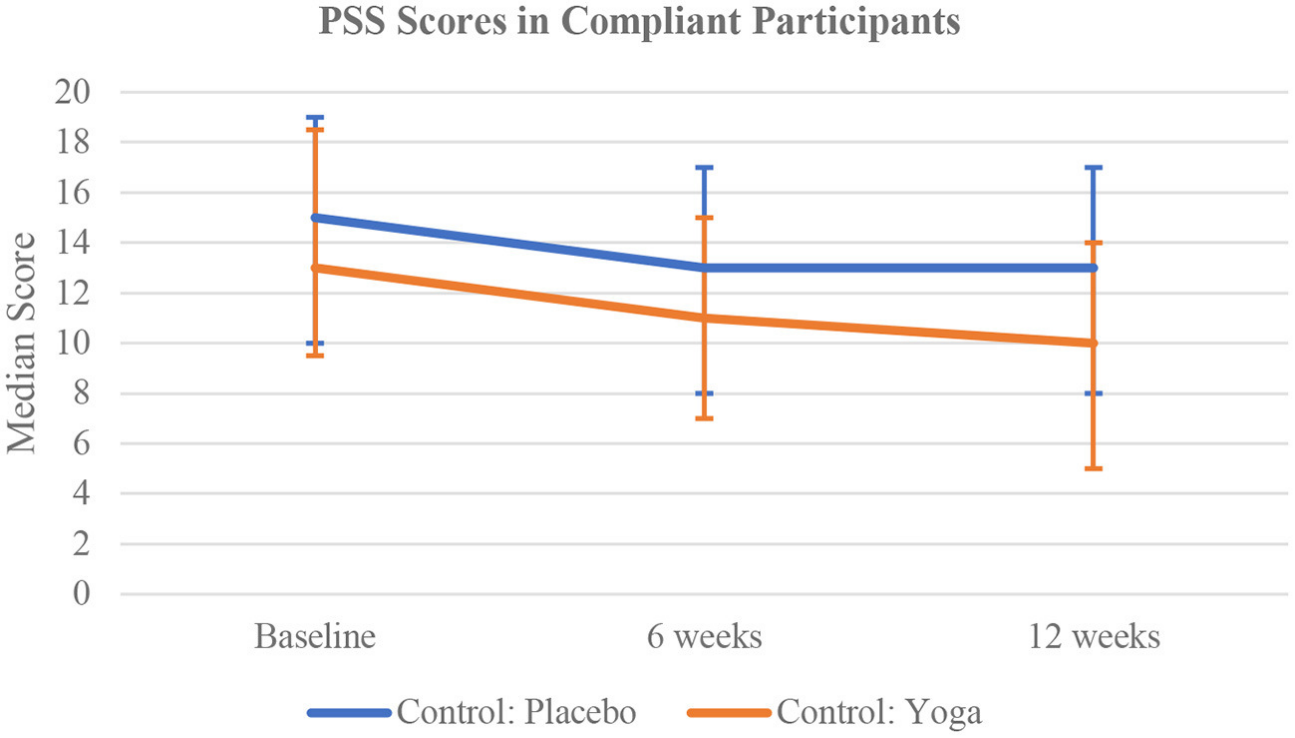
Time trend comparison of PSS Scores in the compliant participants of non-meditator cohort at all three time points.

Compared to the placebo comparators, the active comparators had lower median PSS scores at 6 weeks [11 (IQR 7–15) vs. 13 (IQR 8–17), *p* = 0.082] and at 12 weeks [10 (IQR 5–14) vs. 13 (IQR 8–17), *p* = 0.017] (Figure 5).

**Figure 6:**
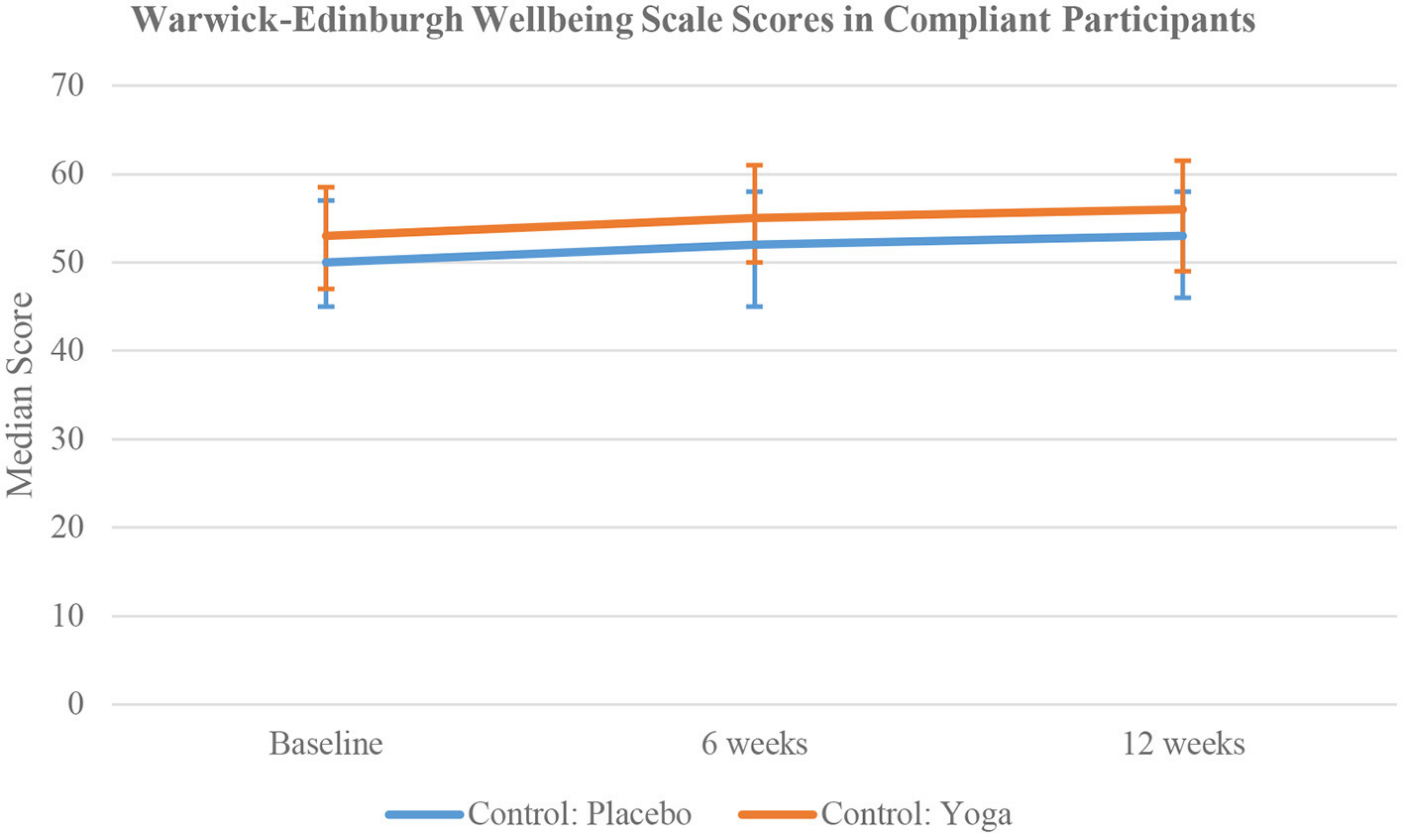
Time trend comparison of WEMWBS Scores in the compliant participants of non-meditator cohort at all three time points.

The active comparator group had higher median wellbeing scores at 6 weeks (*p* = 0.048), and 12 weeks (*p* = 0.046), and higher median joy at 6 weeks (*p* = 0.029), compared to the placebo comparator group (Figure 6). Other measures such as anxiety, depression, resilience, and mindfulness were similar between these two groups at 6 and 12 weeks (Table 4).

**Table 4:** Compliant participant comparison in the comparator group only (at 6 week and 12 week).

	**Week 6**			**Week 12**		
**Scores**	**Active** **comparator** **(*n* = 80)**	**Placebo** **comparator** **(*n* = 100)**	***P*** **-Value**	**Active** **comparator** **(*n* = 80)**	**Placebo** **comparator** **(*n* = 100)**	***P*** **-Value**
**Measures of negative affect** ^a^						
PSS score, median (IQR)	(*n* = 78)^d^	(*n* = 97)	0.082	(*n* = 73)	(*n* = 95)	0.017
	11 (7, 15)	13 (8, 17)		10 (5, 14)	13 (8, 17)	
PHQ anxiety score, No. (%)	(*n* = 78)	(*n* = 97)	0.80	(*n* = 72)	(*n* = 93)	0.82
None	73 (93.6)	92 (94.9)		67 (93.1)	85 (91.4)	
Mild	4 (5.1)	4 (4.1)		4 (5.6)	7 (7.5)	
Moderate	1 (1.3)	1 (1.0)		1 (1.4)	1 (1.1)	
PHQ depression score, No. (%)	(*n* = 78)	(*n* = 97)	0.34	(*n* = 72)	(*n* = 93)	1.0
None	72 (92.3)	93 (95.9)		70 (97.2)	90 (96.8)	
Mild	6 (7.7)	4 (4.1)		2 (2.8)	3 (3.2)	
Moderate	0 (0.0)	0 (0.0)		0 (0.0)	0 (0.0)	
**Measures of positive affect** ^b^						
Warwick-Edinburgh wellbeing scale score, median (IQR)	(*n* = 78)	(*n* = 97)	0.048	(*n* = 72)	(*n* = 93)	0.046
	55 (50, 61)	52 (45, 58)		56 (49, 61.5)	53 (46, 58)	
DPES score, median (IQR)	(*n* = 78)	(*n* = 97)	0.029	(*n* = 72)	(*n* = 94)	0.32
	5.5 (4.5, 6)	5 (4.2, 5.8)		5.3 (4.5, 6)	5.2 (4.3, 6)	
BRS score, median (IQR)	(*n* = 78)	(*n* = 97)	0.56	(*n* = 72)	(*n* = 94)	0.56
	2 (1.8, 2.2)	2 (1.8, 2.2)		2 (1.8, 2.2)	2 (2, 2.2)	
**Mindfulness scores** ^c^						
MAAS score, median (IQR)	(*n* = 78)	(*n* = 97)	0.099	(*n* = 72)	(*n* = 94)	0.087
	4.8 (4, 5.4)	4.4 (3.6, 5.2)		5 (4, 5.5)	4.8 (3.8, 5.2)	
**COVID positive participants only**						
Post-Traumatic growth inventory score, median (IQR)	(*n* = 1)	(*n* = 1)	N/A	(*n* = 1)	(*n* = 2)	1.0
	56 (56, 56)	54 (54, 54)		48 (48, 48)	52 (28, 76)	

a *Decline in scores suggests successful impact of meditation practices,*

b *Increase in scores suggests successful impact of meditation practices,*

c *increase in scores suggests successful impact of meditation practices,*

d *(n = X) represents the number of patients with this score in the specific group at the specific time point*.

The period prevalence rates of COVID-19 between May 21 and Jun 21 among the active comparators, placebo comparators, and yoga practitioners are 0.4, 0.6, and 0.5%, respectively. The rates between July 5 and August 5 are 0.5, 0.4, and 0.3%, respectively. The rates between Aug 15 and Sep 15 were 0.6, 2.3, and 1.1%, respectively. At baseline, 19 (90.5%) of yoga practitioners with COVID-19 reported symptoms of either fever or shortness of breath compared to 2 (40%) of the active comparators and 4 (57.1%) of the placebo comparators (*p* = 0.023). There were no other significant differences in the frequency of symptoms reported between the groups at any other time point. There was no significant difference in the duration of symptoms reported nor in the Post Traumatic Growth Inventory Scores between groups at any time point.

#### Compliance

Compliance is defined as 3 days of activity each week for at least 3 weeks from baseline to 6 weeks, or a minimum of 6 weeks from baseline to 12 weeks. Based on the responses collected from the weekly updates, seasoned yoga practitioners completed their activity for an average of 6.1 days per week (SD:1.5), while active comparator arm only performed Simha Kriya for an average of 3.3 days per week (SD:3.0) and placebo comparator arm performed their chosen activity for an average of 3.8 days per week (SD:2.9) (Refer to Supplementary Tables B–G in Supplementary Materials).

Eighty of the 1,177 active comparators and 100 of the 1,161 placebo comparators were found to be compliant for the full 12 weeks. At baseline, there were no significant differences in the scores between the compliant active and placebo comparators. At 6 weeks, the active comparators had higher median wellbeing [55 (IQR 50–61) vs. 52 (IQR 45–58), *p* = 0.048] and median joy scores [5.5 (IQR 4.5–6) vs. 5 (IQR 4.2–5.8), *p* = 0.029]. compared to the placebo comparators. At 12 weeks, the active comparators had higher wellbeing [56 (IQR 49–61.5) vs. 53 (IQR 46–58), *p* = 0.046] and joy scores [5.5 (IQR 4.5–6) vs. 5 (IQR 4.2–5.8), *p* = 0.029]. compared to the placebo comparators. At 12 weeks, the active comparators also had lower median PSS scores compared to the placebo group with 10 [IQR 5–14] and 13 [IQR 8–17], respectively (*p* = 0.017) (Table 4).

Between baseline and 6 weeks, the median difference in PSS scores among both the compliant active and placebo comparators was a three-unit [IQR −5–0] decrease (*p* < 0.0001). There was a 1.5-unit [IQR −1–5] increase in wellbeing scores among the active comparators (*p* = 0.0008), and a two-unit [IQR −2–5] increase among the placebo comparators (*p* = 0.0042). For joy scores, there was a zero-unit [IQR −0.2–0.5] change among the active comparators (*p* = 0.035), but no significant change among the placebo comparators.

Between 6 and 12 weeks, the median difference in PSS scores among the compliant active comparators was a one-unit [IQR −5–2] decrease (*p* = 0.025). However, there was no significant change among the placebo comparators. Joy scores increased by 0.2 units [IQR −0.3–0.5] among the placebo comparators (*p* = 0.037), but there was no significant change among the active comparators. Mindfulness awareness (MAAS) scores increased by 0.2 units [IQR −0.2–0.6] between 6 and 12 weeks among the active comparators (*p* = 0.0033) but not the placebo comparators.

## Discussion And Conclusion

Seasoned yoga practitioners had better psychological status compared to the rest of the study population at all-time points. Lower levels of stress in seasoned yoga practitioners have also been documented in studies conducted by Tyagi et al. (29) and Peterson et al. (30). Significant lowering in levels of depression in yoga practitioners appeared in results of a randomized control trial study conducted by Prathikanti et al. (20).

Furthermore, when subjected to a short 3-min online guided breathing exercise (Simha Kriya), the control arm participants demonstrated significant changes in their perceived stress. The active comparator arm reported lower levels of stress at weeks 6 and 12. This result demonstrates the positive effect of a 3-min breathing and meditation practice on diminishing stress levels. Our findings are comparable to Doria et al. (31) where a yoga practice that includes a specific breathing technique reduced stress levels in patients suffering from generalized anxiety disorder. Vinchurkar et al. (32) found that short periods of yoga and meditation improved mental health. Other health benefits reported by Peterson et al. (30) include sleep quality improvement, higher levels of focus and attention, and physiological benefits such as stabilizing the cardiac autonomic nervous system in yoga practitioners. These studies emphasize the importance of a short period of exposure to mindfulness practices that can result in improved mental health in participants.

Next, a closer look at compliant participant scores in the cohort with brief exposure to yoga intervention (Simha Kriya) revealed a sustained improvement in PSS scores at week 6 and week 12 compared to non-compliant participants. These findings agree with those reported by Chang et al. (33) in a waitlisted RCT in college students during the pandemic. They reported that consistent practice of yoga for 3 or more times per week resulted in significant changes in stress, anxiety, depression, wellbeing, resilience, positive & negative affect scores. Sadhasivam et al. (34) found similar results in a study conducted wherein study participants of a four-day yoga retreat experienced improved focus, happiness, and positive wellbeing with reduced depression and anxiety. Scores increased immediately after the retreat compared with participants' baseline values assessed 2 weeks before the program *(p* < 0.001). All improvements were sustained 1 month after the program. Blood tests from participants (*n* = 142) also showed increased endocannabinoid levels (lipid mediators associated with enhanced mood and reduced anxiety/depression) as well as a brain-derived neurotrophic factor suggesting a role for these biomarkers in the underlying mechanism of yoga's protective effects.

With implementation of social distancing and work from home approaches to curb the spread of COVID-19 pandemic, telemedicine has become a cornerstone in healthcare delivery approaches (35, 36). Meditation and yoga are optimal choices for complementary health practices for promotion of mental and physical wellbeing (37). It is important to recognize that the current study has successfully demonstrated the scalability and accessibility of Simha Kriya as an intervention. While several studies are now studying the impact of remote administration of the mindfulness-based intervention, only a few have been able to successfully implement them (38, 39).

### Study Strength

The study draws its strength from a large number of participants enrolled in the study. Small sample sizes and relatively self-selected population enrolling into mindfulness-based research studies often affect the scalability and generalizability of the studies' results. Furthermore, the complex study design enabled the flexibility of simply observing the seasoned yoga practitioners in their typical practice while simultaneously generating and testing a hypothesis on the effects of a short breathing practice on novice practitioners. The use of validated neuro-psychological scales lent validity to the self-reported survey responses and helped establish an association between yoga and mental health outcomes. Finally, as previously mentioned, despite being employed in a sub-group of control participants i.e., active comparator group, the study team was able to remotely deliver and provide a brief yet effective breathing practice in times of need.

### Study Limitations

A limitation of our study is that we could not randomize all study participants. As the investigation was performed during peak COVID-19 infections in the U.S., it would have been unethical to advise routine yoga practitioners to forego their practices for the sake of the study's internal validity. In order to account for the various confounding variables on the data collected, the study team undertook the following measures:

1. Actively match for age, gender, and region between the two cohorts of seasoned yoga practitioners and controls

2. Treating and analyzing the data collected from seasoned practitioners as an observational arm, and

3. Finally, collecting detailed information on intervention practiced, frequency of interventions, and duration of practice and accounted for these details in analysis phase.

The study team acknowledges that the two cohorts were not identical and that seasoned yoga practitioners had greater exposure to the mindfulness practices with an advantage of time and experience than the controls. As Davidson and Kaszniak (40) eloquently offer in their review; estimating mindfulness is complex by virtue of several confounding variables (e.g., mindfulness practice time, style of practice, home vs. retreat practice, formal vs. informal practice, age, and cultural variations etc.). These variables lead to variation in expectations from seasoned vs. novice practitioner's mindfulness quality and experience. Quantification of these variables is not possible with use of self reports alone.

Another limitation that can be identified is that only Isha School of yoga practitioners were invited to participate in this study, introducing an element of selection bias. However, since the comparator cohort composed of novice practitioners was subjected to randomization, selection bias did not truly impact the study's reported outcome measures. Seasoned yoga practitioners were treated as an observational cohort while the comparator arm participants: who are recruited by snowball sampling technique and have no prior meditation or yoga experience, were introduced to either intervention or control group activities based on the group they are allocated.

Lastly, many participants did not complete the study, and the participant attrition rates were high in all cohorts. The attrition rates amounted to ~50% at each time point which is fairly consistent with the wide range for attrition reported in the literature, i.e., 8–60% (41, 42). The difficult time faced by the participants during the pandemic and the usual reasons could be a contributing cause.

### Future Directions

The study team identifies a lack of objective physiological markers provided by wearable health-tracking devices as a limitation of the current study. The study team could not introduce data collection from these devices due to financial and time constraints when the study was introduced. Additionally, restrained physical communication and work from home added another layer of complexity in data collection from such devices. These were also the reasons why we refrained from any lab collections for the study. However, the study team aims at incorporating data points from these devices as a compliance marker in upcoming studies to make a more robust comparison.

With this study, the study team demonstrated that seasoned yoga practitioners had better psychological status compared to the rest of the study population at all-time points thereby signaling the protective effects of yoga practice, especially during the uncertain times of a pandemic. Further adherence to a brief 3-min breathing practice for as less as 3 days/week resulted in sustained improvements in stress and mental wellbeing of study participants. This offers an opportunity for providing Simha kriya as remotely delivered, accessible practice to all who suffer from COVID-19 as an adjunct therapy. Further clinical efficacy trails are warranted to establish the true impact of such breathing techniques.

## Conclusion

We provide evidence that routine yoga practice during the COVID-19 pandemic did reduce stress and enhanced wellbeing in study participants who were exposed to some form of yoga activity during the 12-week study duration.

## Data Availability Statement

The raw data supporting the conclusions of this article will be made available by the authors, without undue reservation.

## Ethics Statement

The studies involving human participants were reviewed and approved by Commitee on Clinical Investigations at Beth Israel Deaconess Medical Center. The patients/participants provided their written informed consent to participate in this study.

## Author Contributions

BS, PU, and LN conceptualized the study's design. SN, TK, PM, and AS conducted data collection. PU, LN, and LK carried out the data analysis. PU did project administration/supervision. LN and LK verified the underlying data. All authors contributed to the writing, review, editing, confirm that they had full access to all the data in the study, and accept responsibility to submit for publication.

## Conflict of Interest

The authors declare that the research was conducted in the absence of any commercial or financial relationships that could be construed as a potential conflict of interest.

## Publisher's Note

All claims expressed in this article are solely those of the authors and do not necessarily represent those of their affiliated organizations, or those of the publisher, the editors and the reviewers. Any product that may be evaluated in this article, or claim that may be made by its manufacturer, is not guaranteed or endorsed by the publisher.
