# Supplementary material for: Perceived Stress, Resilience, and Wellbeing in Seasoned Isha Yoga Practitioners Compared to Matched Controls During the COVID-19 Pandemic
Source: Front Public Health. 2022 Jul 29;10:813664. doi: 10.3389/fpubh.2022.813664 (PMC9372270; doi:10.3389/fpubh.2022.813664)
Supplement: Supplementary file 1 [file Data_Sheet_1.docx]

**Supplementary Material**

**Supplementary figures: Geo-chart for week 6 distribution**


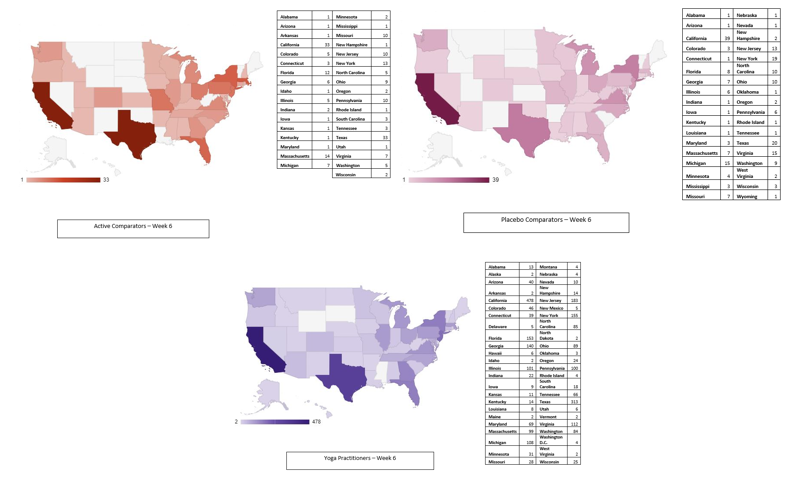


**Supplementary figures: Geo-chart for week 12 distribution**


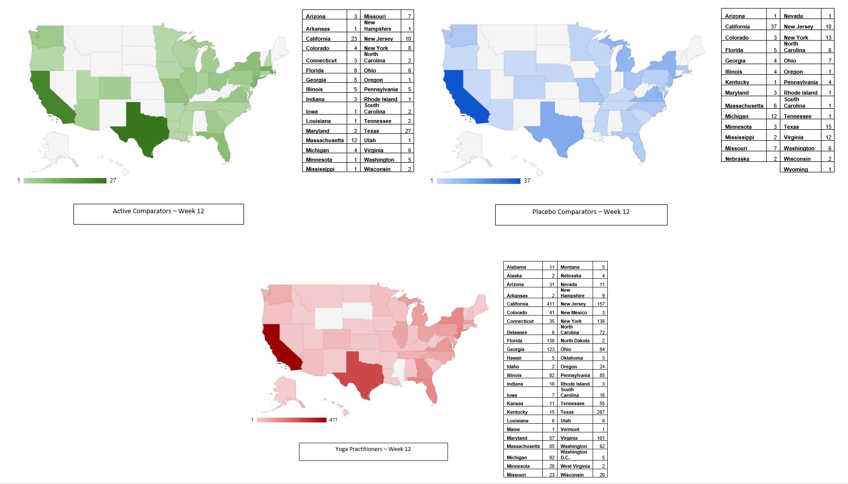


**Table A: Primary outcome – regression analysis (in supplementary section)**

|  | **Baseline** | | **Week 6** | | **Week 12** | |
| --- | --- | --- | --- | --- | --- | --- |
|  | **RR (95 % CI)** | **P-Value** | **RR (95 % CI)** | **P-Value** | **RR (95 % CI)** | **P-Value** |
| Group  Placebo Comparators  Active Comparators  Yoga Practitioners | Ref.  1·01 (0·97 – 1·05)  0·69 (0·67 - 0·71) | -----  0·61  <0·0001 | Ref.  0·93 (0·85 – 1·03)  0·70 (0·65 - 0·75) | -----  0·16  <0·0001 | Ref.  0·89 (0·79 – 1·0)  0·71 (0·65 – 0·77) | -----  0·059  <0·0001 |
| Age | 1·0 (0·99 – 1·0) | <0·0001 | 0·99 (0·99 – 1·0) | <0·0001 | 0·99 (0·99 – 0·99) | <0·0001 |
| Region  Midwest  South East  South West  West  North East | 0·99 (0·96 – 1·03)  1·01 (0·98 – 1·05)  1·02 (0·98 – 1·06)  1·01 (0·98 – 1·04)  Ref. | 0·65  0·56  0·23  0·57  ----- | 1·03 (0·97 – 1·10)  1·03 (0·97 – 1·09)  1·06 (0·99 – 1·14)  1·03 (0·97 – 1·09)  Ref. | 0·37  0·39  0·081  0·35  ----- | - 1. (0·95 – 1·11)   1·08 (1·0 – 1·15)  1·10 (1·01 – 1·19)  1·04 (0·97 – 1·11)  Ref. | 0·51  0·041  0·028  0·30  ----- |
| Employment Status  Employed Full Time  Employed Part Time (or Self-Employed, Contingent worker)  Not employed/Laid off  Retired  Other (Disabled, Student, Military Service) | Ref.  1·01 (0·98 – 1·05)  1·05 (1·01 – 1·09)  0·91 (0·85 – 0·97)  1·06 (1·01 – 1·11) | -----  0·45  0·0070  0·0035  0·016 | Ref.  1·0 (0·95– 1·06)  1·09 (1·02 – 1·16)  0·95 (0·86 – 1·05)  1·07 (0·99 – 1·17) | -----  0·94  0·0093  0·33  0·099 | Ref.  0·99 (0·93 – 1·06)  1·12 (1·04 – 1·2)  0·97 (0·87 – 1·08)  1·07 (0·98 – 1·18) | -----  0·80  0·0032  0·59  0·15 |

^a^ Model adjusted for age, region, and employment status

**Table B: Frequency distribution of Seasoned Yoga Practitioners Group Practice (in supplementary section)**

| **Week** | **1 Day** | **2 Days** | **3 Days** | **4 Days** | **5 Days** | **6 Days** | **7 Days** |
| --- | --- | --- | --- | --- | --- | --- | --- |
| Week 1 (n=2519) | 119 | 50 | 92 | 131 | 243 | 293 | 1591 |
| Week 2  (n=2692) | 74 | 41 | 102 | 150 | 295 | 356 | 1674 |
| Week 3  (n=2599) | 55 | 37 | 90 | 149 | 292 | 352 | 1624 |
| Week 4  (n=2550) | 59 | 46 | 115 | 141 | 277 | 350 | 1562 |
| Week 5  (n=2388) | 43 | 50 | 94 | 126 | 249 | 353 | 1473 |
| Week 6  (n=2409) | 39 | 39 | 78 | 115 | 266 | 336 | 1536 |
| Week 7  (n=2221) | 41 | 43 | 81 | 124 | 232 | 334 | 1366 |
| Week 8  (n=2165) | 46 | 47 | 82 | 112 | 244 | 303 | 1331 |
| Week 9  (n=2118) | 49 | 40 | 82 | 112 | 227 | 300 | 1308 |
| Week 10  (n=2085) | 47 | 46 | 88 | 112 | 229 | 289 | 1274 |
| Week 11  (n=1993) | 49 | 36 | 82 | 118 | 217 | 288 | 1203 |
| Week 12  (n=1859) | 40 | 42 | 63 | 103 | 183 | 267 | 1161 |

**Table C: Seasoned Yoga Practitioners Group Practice weekly average & medians (in supplementary section)**

| **Week** | **n** | **Mean ± SD** | **Median (IQR)** |
| --- | --- | --- | --- |
| **Week 1** | 2519 | 6 (1.7) | 7 (5, 7) |
| **Week 2** | 2692 | 6.1 (1.5) | 7 (6, 7) |
| **Week 3** | 2599 | 6.1 (1.4) | 7 (6, 7) |
| **Week 4** | 2550 | 6.1 (1.5) | 7 (5, 7) |
| **Week 5** | 2388 | 6.1 (1.4) | 7 (6, 7) |
| **Week 6** | 2409 | 6.2 (1.4) | 7 (6, 7) |
| **Week 7** | 2221 | 6.1 (1.4) | 7 (6, 7) |
| **Week 8** | 2165 | 6.1 (1.5) | 7 (6, 7) |
| **Week 9** | 2118 | 6.1 (1.5) | 7 (6, 7) |
| **Week 10** | 2085 | 6.1 (1.5) | 7 (5, 7) |
| **Week 11** | 1993 | 6.1 (1.5) | 7 (5, 7) |
| **Week 12** | 1859 | 6.1 (1.5) | 7 (6, 7) |

**Table D: Frequency distribution of Active Comparator Group Practice (in supplementary section)**

| **week** | **I did not practice activities this week** | **1 Day** | **2 Days** | **3 Days** | **4 Days** | **5 Days** | **6 Days** | **7 Days** |
| --- | --- | --- | --- | --- | --- | --- | --- | --- |
| **Week 1**  **(n=314)** | 113 | 24 | 19 | 19 | 19 | 26 | 24 | 70 |
| **Week 2**  **(n=256)** | 85 | 13 | 15 | 17 | 17 | 22 | 18 | 69 |
| **Week 3**  **(n=224)** | 78 | 8 | 7 | 14 | 16 | 20 | 19 | 62 |
| **Week 4**  **(n=203)** | 70 | 5 | 13 | 8 | 11 | 18 | 21 | 57 |
| **Week 5**  **(n=174)** | 61 | 10 | 5 | 9 | 9 | 12 | 18 | 50 |
| **Week 6**  **(n=168)** | 64 | 4 | 18 | 9 | 11 | 14 | 7 | 41 |
| **Week 7**  **(n=165)** | 71 | 3 | 6 | 11 | 3 | 14 | 10 | 47 |
| **Week 8**  **(n=144)** | 57 | 2 | 6 | 7 | 7 | 15 | 9 | 41 |
| **Week 9**  **(n=151)** | 62 | 2 | 6 | 8 | 10 | 11 | 8 | 44 |
| **Week 10**  **(n=136)** | 53 | 3 | 7 | 2 | 8 | 15 | 5 | 43 |
| **Week 11**  **(n=134)** | 57 | 2 | 5 | 6 | 8 | 9 | 12 | 35 |
| **Week 12**  **(n=112)** | 50 | 3 | 8 | 9 | 5 | 7 | 7 | 23 |

**Table E: Active Comparator Group Practice weekly average & medians (in supplementary section)**

| **Week** | **n** | **Mean ± SD** | **Median (IQR)** |
| --- | --- | --- | --- |
| **Week 1** | 324 | 3.1 (2.9) | 3 (0, 6) |
| **Week 2** | 264 | 3.4 (2.9) | 3 (0, 7) |
| **Week 3** | 237 | 3.5 (3.0) | 4 (0, 7) |
| **Week 4** | 215 | 3.5 (3.0) | 4 (0, 7) |
| **Week 5** | 185 | 3.5 (3.0) | 4 (0, 7) |
| **Week 6** | 219 | 3.0 (2.9) | 2 (0, 6) |
| **Week 7** | 174 | 3.1 (3.1) | 3 (0, 7) |
| **Week 8** | 151 | 3.3 (3.0) | 3.5 (0, 7) |
| **Week 9** | 160 | 3.2 (3.0) | 3 (0, 7) |
| **Week 10** | 143 | 3.4 (3.1) | 4 (0, 7) |
| **Week 11** | 143 | 3.2 (3.0) | 3 (0, 7) |
| **Week 12** | 163 | 2.7 (2.9) | 2 (0, 6) |

**Table F: Frequency distribution of Placebo Comparator Group Practice (in supplementary section)**

| **week** | **I did not practice activities this week** | **1 Day** | **2 Days** | **3 Days** | **4 Days** | **5 Days** | **6 Days** | **7 Days** |
| --- | --- | --- | --- | --- | --- | --- | --- | --- |
| **Week 1**  **(n=324)** | 90 | 13 | 17 | 20 | 29 | 33 | 25 | 97 |
| **Week 2**  **(n=258)** | 81 | 7 | 15 | 20 | 14 | 24 | 27 | 70 |
| **Week 3**  **(n=215)** | 50 | 12 | 9 | 15 | 13 | 17 | 23 | 76 |
| **Week 4**  **(n=182)** | 48 | 2 | 5 | 12 | 12 | 23 | 13 | 67 |
| **Week 5**  **(n=178)** | 51 | 3 | 3 | 17 | 10 | 19 | 18 | 57 |
| **Week 6**  **(n=191)** | 52 | 6 | 12 | 15 | 15 | 20 | 10 | 61 |
| **Week 7**  **(n=167)** | 46 | 3 | 6 | 10 | 15 | 15 | 13 | 59 |
| **Week 8**  **(n=170)** | 54 | 3 | 10 | 9 | 8 | 17 | 7 | 62 |
| **Week 9**  **(n=165)** | 50 | 1 | 8 | 11 | 15 | 14 | 13 | 53 |
| **Week 10**  **(n=157)** | 53 | 5 | 7 | 9 | 12 | 13 | 9 | 49 |
| **Week 11**  **(n=147)** | 50 | 4 | 2 | 12 | 10 | 8 | 9 | 52 |
| **Week 12**  **(n=129)** | 43 | 4 | 10 | 13 | 8 | 15 | 4 | 32 |

**Table G: Placebo Comparator Group Practice weekly average & medians (in supplementary section)**

| **Week** | **n** | **Mean ± SD** | **Median (IQR)** |
| --- | --- | --- | --- |
| **Week 1** | 327 | 3.8 (2.9) | 4 (0, 7) |
| **Week 2** | 266 | 3.6 (2.9) | 4 (0, 7) |
| **Week 3** | 222 | 4.1 (2.9) | 5 (1, 7) |
| **Week 4** | 188 | 4.2 (2.9) | 5 (0, 7) |
| **Week 5** | 183 | 3.9 (2.9) | 5 (0, 7) |
| **Week 6** | 229 | 3.8 (2.9) | 4 (0, 7) |
| **Week 7** | 176 | 4.0 (2.9) | 5 (0, 7) |
| **Week 8** | 174 | 3.8 (3.0) | 5 (0, 7) |
| **Week 9** | 167 | 3.8 (2.9) | 4 (0, 7) |
| **Week 10** | 160 | 3.5 (3.0) | 4 (0, 7) |
| **Week 11** | 149 | 3.7 (3.0) | 4 (0, 7) |
| **Week 12** | 174 | 3.2 (2.8) | 3 (0, 6) |
